# Doença de Fabry de Início Tardio: Desafios Diagnósticos e Evolução Clínica

**DOI:** 10.36660/abc.20250554

**Published:** 2026-03-26

**Authors:** Hernán Patricio García Mejía, Heleuterio da Conceição Nicolau Madogolele, Manuela Cristina Ribeiro Dias Barroso, Matheus Carvalho Alves Nogueira, Kevin Rafael de Paula Morales, Cristhian Espinoza Romero, Luciano Nastari, Fábio Fernandes

**Affiliations:** 1 Instituto do Coração do Hospital das Clínicas da Faculdade de Medicina da Universidade de São Paulo São Paulo SP Brasil Instituto do Coração do Hospital das Clínicas da Faculdade de Medicina da Universidade de São Paulo, São Paulo, SP – Brasil

**Keywords:** Doença de Fabry, Hipertrofia Ventricular Esquerda, Cardiomiopatias, Distúrbios do Sistema de Condução Cardíaco

## Introdução

O acometimento simultâneo cardíaco e renal pode ser resultado de diversas condições sistêmicas, como hipertensão, doenças autoimunes, doenças infiltrativas e síndromes hereditárias. A doença de Fabry (DF) é uma desordem genética ligada ao cromossomo X, causada pela deficiência ou ausência da enzima α-galactosidase A, levando ao acúmulo progressivo de glicoesfingolipídeos, especialmente globotriaosilceramida (Gb3), em diversos tecidos.^1^ Esse acúmulo de Gb3 promove disfunções multissistêmicas, com manifestações clínicas heterogêneas que variam de acordo com o sexo, o grau de atividade enzimática residual e a forma de apresentação, clássica ou não clássica.^2,3^

A incidência estimada da DF varia entre 1 em 40.000 a 1 em 117.000 indivíduos no mundo.^2^ No Brasil, a epidemiologia da DF permanece pouco definida, devido à raridade da condição e à subnotificação dos casos, o que reforça o valor de relatos clínicos como o presente.

Embora a forma clássica da doença se manifeste precocemente, a variante de apresentação tardia é frequentemente subdiagnosticada e se caracteriza por envolvimento predominantemente cardíaco, renal ou neurológico em pacientes adultos, geralmente do sexo masculino. No contexto cardiológico, a manifestação mais comum é o fenótipo de miocardiopatia hipertrófica não obstrutiva, frequentemente indistinguível de outras causas sem realizar uma investigação específica.

## Relato de caso

Paciente masculino, 71 anos, encaminhado pela equipe de Nefrologia para investigação de hipertrofia ventricular esquerda (HVE). Antecedentes patológicos: hipertensão arterial sistêmica (HAS) de longa data, doença renal crônica (DRC) dialítica desde fevereiro de 2018 e infarto agudo do miocárdio.

História familiar: morte súbita de uma irmã aos 36 anos, pai falecido por cardiopatia não especificada aos 68 anos e mãe aos 60 anos de causa ignorada. Uma filha viva, saudável.

**Figura 1:**
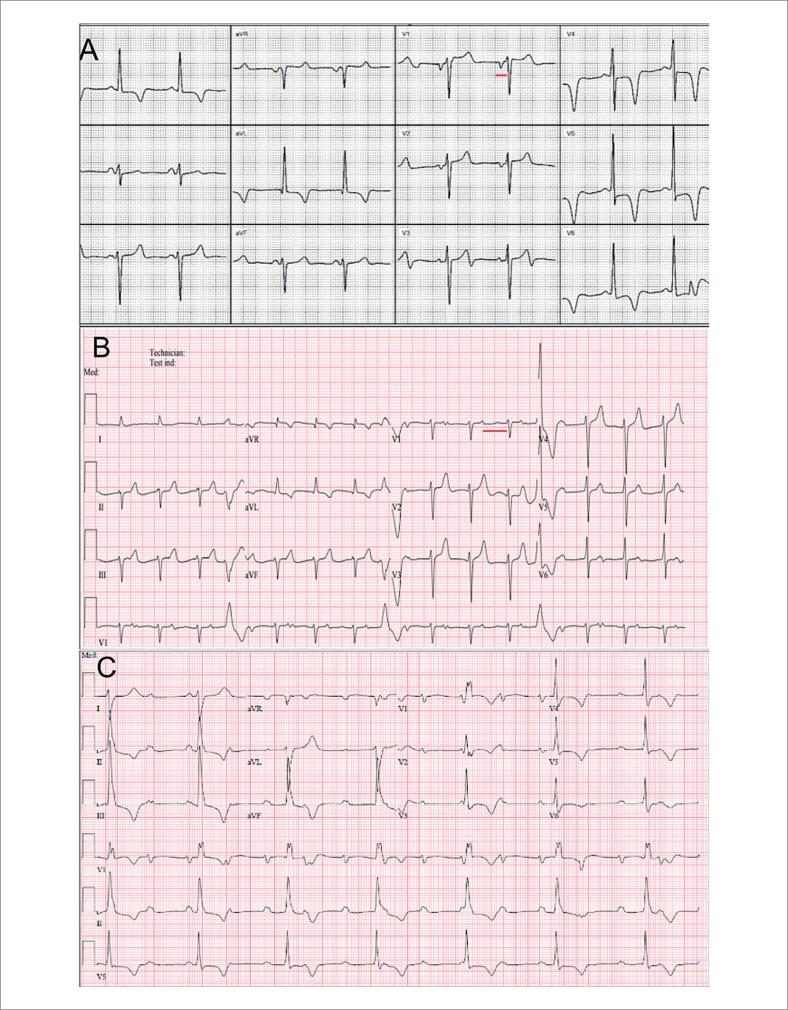
A) Eletrocardiograma mostrando ritmo sinusal, intervalo PR curto (88 ms), bloqueio anterofascicular esquerdo e hipertrofia ventricular esquerda. B) Eletrocardiograma mostrando ritmo sinusal, bloqueio atrioventricular de primeiro grau (380 ms), contrações ventriculares prematuras, bloqueio antero fascicular esquerdo. C) Eletrocardiograma mostrando padrão de bloqueio de ramo direito e bloqueio atrioventricular de segundo grau Mobitz II.

**Figura 2:**
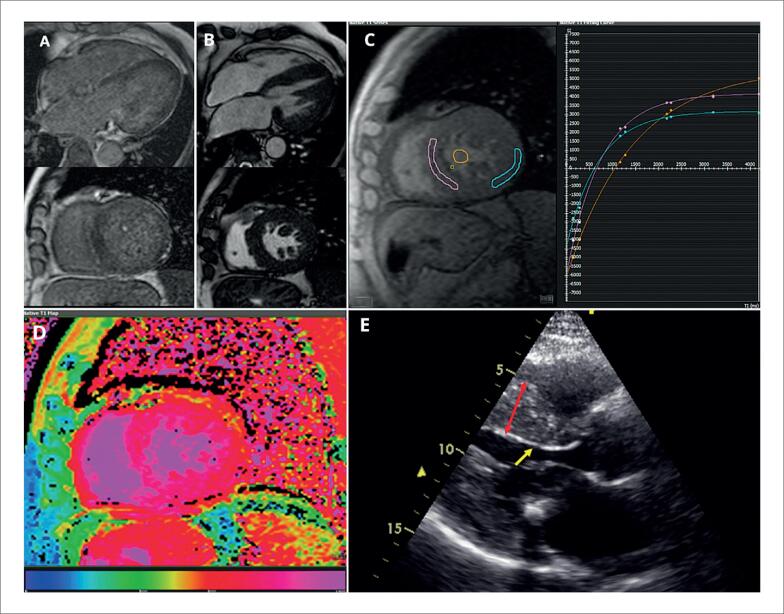
A) Imagens de realce tardio miocárdico obtidas após administração de gadolínio, sem evidência de fibrose miocárdica. B) Sequência cine por ressonância magnética utilizando técnica SSFP (steady-state free precession), demonstrando aumento concêntrico da espessura do ventrículo esquerdo. C) Mapa T1 nativo adquirido por sequência MOLLI (Modified Look-Locker Inversion recovery), demonstrando valores de T1 de 1119 ms no septo interventricular e 909 ms na parede lateral, a partir de regiões de interesse (ROIs). D) Mapa paramétrico T1 nativo em corte eixo curto médio, com codificação em escala de cores, demonstrando visualmente predominância de valores elevados na parede septal. E) Ecocardiografia transtorácica mostrando espessura do septo interventricular de 17mm (seta vermelha), e a presença do sinal binário (seta amarela).

O eletrocardiograma (ECG) inicial (2018) evidenciava ritmo sinusal, intervalo PR curto (88 ms), bloqueio divisional ântero-superior esquerdo e sinais de HVE (Figura 1-A). A ecocardiografia transtorácica mostrou HVE importante, septo interventricular 17 mm, parede posterior 15 mm, padrão não obstrutivo (Figuras 2-A e 2-B). O cateterismo identificou gradiente intraventricular de 26 mmHg e doença coronariana triarterial.

A ressonância magnética cardíaca (RMC) confirmou HVE simétrica, FEVE preservada (VE: 74%, VD: 62%), sem realce tardio, porém com aumento do volume extracelular (40%; VR <30%) e T1 nativo elevado (Valor de referência (VR): 1119 ms; VR 968 ± 21 ms), além de área inferolateral com T1 baixo (VR: 898 ms).

Diante do quadro de hipertrofia concêntrica importante, foram considerados diagnósticos diferenciais como amiloidose cardíaca e DF. A eletroforese de proteínas séricas e urinárias mostrou padrão normal, com cadeias leves kappa e lambda dentro dos valores de referência. A dosagem enzimática revelou atividade reduzida de α-galactosidase A: 0,88 mcmol/L/h (VR: 1,68–13,63 mcmol/L/h) e dosagem da LysoGb3 <0,5 ng/mL (VR: <2,0 ng/mL), e a análise genética identificou uma mutação em homozigose no gene GLA (c.870G>C; p.Met290Ile), variante rara descrita de forma limitada na literatura, associada a fenótipo tardio e predomínio de manifestações cardíacas, confirmando o diagnóstico de DF de apresentação tardia. O paciente iniciou terapia de reposição enzimática (TRE) com agalsidase beta, 1 mg/kg quinzenal, com resposta clínica inicial satisfatória.

A filha do paciente também apresentou a mesma mutação em heterozigose, com atividade enzimática dentro da normalidade (2,44 mcmol/L/h). Foi realizado aconselhamento genético aos demais familiares de primeiro grau, com orientação para rastreamento clínico e molecular.

Após 4 anos de TRE, houve piora da classe funcional, aumento dos marcadores como BNP/troponina e palpitações. O ECG mostrou prolongamento progressivo do PR (88 → 380 ms), bloqueio atrioventricular (BAV) de primeiro grau, extrassístoles ventriculares frequentes (Figura 1-B). Evoluindo posteriormente em junho de 2025 com episódios sincopais do tipo "desliga-liga". O monitoramento eletrocardiográfico contínuo confirmou BAV de segundo grau tipo Mobitz II, sendo então indicado e realizado implante de marca-passo definitivo (Figura 1-C).

## Discussão

A DF é uma condição hereditária ligada ao cromossomo X, caracterizada pelo acúmulo de glicoesfingolipídeos, sobretudo globotriaosilceramida (Gb3), em diferentes tecidos, incluindo miocárdio, rim, endotélio e sistema de condução cardíaco.^1^ Esse acúmulo resulta em um espectro clínico heterogêneo, desde formas clássicas graves até variantes de início tardio, frequentemente subdiagnosticadas.^1,2^

No Brasil, a epidemiologia da DF ainda não é bem estabelecida devido à raridade, à variabilidade fenotípica e à subnotificação de casos, havendo escassez de dados em registros nacionais. Estudos internacionais relatam prevalência de variantes patogênicas do gene GLA em até 1% dos pacientes com fenótipo de HVE.^3,4^ Um estudo brasileiro recente envolvendo pacientes em hemodiálise identificou variantes patogênicas ou de significado incerto no gene GLA em quase metade da amostra, reforçando a relevância clínica do rastreamento genético nesse contexto e a necessidade de interpretação cuidadosa de variantes raras, como a mutação p.Met290Ile descrita no presente caso.^5^

As mutações clássicas da DF são observadas em cerca de 1:22.000 a 1:40.000 homens, e na forma não clássica em aproximadamente 1:1.000 a 1:3.000 homens e 1:6.000 a 1:40.000 mulheres.^6^ O fenótipo clássico, mais grave, é caracterizado pela ausência ou redução severa da atividade de α-Gal A, acúmulo de Gb3 e início precoce dos sintomas, apresentando-se principalmente na infância e adolescência. Já o fenótipo não clássico, ou de início tardio, apresenta-se na vida adulta, com envolvimento sistêmico incompleto e alguma atividade residual da enzima, como no caso do paciente descrito.

**Figura 3:**
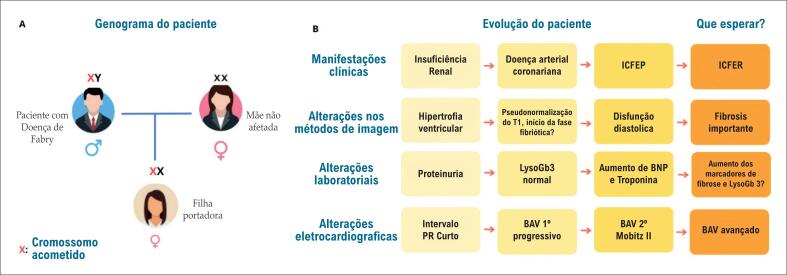
A) Genograma do paciente; B) Evolução das alterações do paciente cronologicamente; ICFEP: insuficiência cardiaca de fração de ejeção preservada; ICFER: insuficiência cardíaca de fração de ejeção reduzida; BAV: bloqueio atrioventricular.

Do ponto de vista estrutural, o acúmulo de Gb3 acomete diversos tipos de células cardíacas, como os cardiomiócitos, células endoteliais, músculo liso, fibras valvares e tecido condutor.^7^ O aumento progressivo de Gb3 nos miócitos leva ao desenvolvimento de HVE e disfunção diastólica. O acometimento dos vasos intramiocárdicos provoca alterações estruturais e funcionais que contribuem para a ocorrência de isquemia miocárdica. Já a presença de fibrose e o comprometimento do sistema de condução cardíaco estão relacionados ao surgimento de distúrbios de ritmo ventricular e alterações na condução elétrica. Em estágios iniciais, pode-se observar intervalo PR encurtado, enquanto BAV e bloqueios de ramo são mais frequentes em fases avançadas. Isso evidencia a progressão dinâmica das alterações (Figura 3).^8,9^ A ausência de dados robustos e estudos específicos sobre risco de morte súbita e arritmias dificulta a estratificação de risco. Uma revisão sistemática publicada em 2017 identificou alguns fatores associados, como idade avançada, sexo masculino, HVE e realce tardio na RMC, mas ainda são necessários estudos mais específicos.^6^

Os métodos diagnósticos incluem exames de imagem como ecocardiografia e RMC, testes de atividade enzimática, testes genéticos, histórico familiar, avaliação clínica e biópsias de tecidos. O diagnóstico diferencial se baseia no espectro das miocardiopatias hipertróficas. Em um estudo recente envolvendo 5.491 pacientes diagnosticados com HVE e/ou cardiomiopatia hipertrófica, a prevalência de variantes patogênicas no gene GLA foi de 0,93% em homens e 0,90% em mulheres.^4^

A RMC e o mapeamento de T1 são ferramentas fundamentais no diagnóstico e no acompanhamento da DF. O mapeamento nativo de T1 mede o tempo de relaxamento longitudinal do tecido miocárdico sem o uso de contraste, auxiliando na caracterização tecidual. Na DF, o acúmulo de Gb3 no miocárdio normalmente leva à redução dos valores nativos de T1. No entanto, o aumento da hipertrofia, a intensificação da fibrose intersticial e a inflamação miocárdica são mecanismos potenciais que explicam o fenômeno de pseudonormalização do T1.^10^ Esse fenômeno, já relatado em estudos anteriores,^11,12^ pode dificultar a interpretação isolada do T1. Portanto, a combinação do mapeamento de T1 com outras modalidades de imagem e avaliações clínicas é essencial para um diagnóstico preciso.^13^

A TRE tem mostrado desacelerar a progressão da doença cardíaca e reduzir a incidência de eventos cardiovasculares. Nos indivíduos com DF de início tardio, deve ser considerada se houver evidência de dano renal, cardíaco ou neurológico, mesmo na ausência de sintomas típicos.^10^ Estudos de longo prazo e registros indicam que a TRE pode retardar a progressão da doença e reduzir eventos cardiovasculares. A TRE alterou significativamente a evolução natural da DF e melhorou a qualidade de vida dos pacientes.^8^

O acometimento simultâneo cardíaco e renal pode decorrer de diversas condições sistêmicas, o que demanda ampla consideração de diagnósticos diferenciais. Entre as causas mais prevalentes destacam-se a cardiopatia e nefropatia hipertensiva, frequentemente coexistentes, tornando-se potenciais fatores de confusão na interpretação do fenótipo.^14,15^ Doenças autoimunes sistêmicas, como lúpus eritematoso sistêmico e vasculites, representam outras importantes etiologias, particularmente na presença de manifestações clínicas e laboratoriais indicativas de inflamação sistêmica.^16^ Doenças infiltrativas, como amiloidose, sarcoidose e hemocromatose, também devem ser consideradas, dada sua capacidade de provocar disfunção miocárdica e lesão renal progressiva.^17^ Além disso, síndromes hereditárias, incluindo DF, doença de Alport e algumas mitocondriopatias, devem ser investigadas.^18^

Por se tratar de um caso único, há limitações inerentes quanto à sua aplicabilidade mais ampla. Mesmo assim, o relato evidencia os desafios diagnósticos impostos pelas comorbidades e a relevância do rastreamento genético.

## Conclusão

A forma não clássica da DF frequentemente se manifesta de maneira insidiosa na vida adulta, com sinais e sintomas atípicos que dificultam seu reconhecimento. Alterações cardíacas, como HVE, disfunção miocárdica e distúrbios do sistema de condução, podem ocorrer mesmo na ausência de manifestações extracardíacas típicas. Além disso, a presença de comorbidades comuns, como HAS e DRC, pode mascarar o quadro clínico e retardar o diagnóstico. Diante disso, torna-se essencial a investigação etiológica aprofundada em casos de miocardiopatia hipertrófica em adultos. A inclusão da DF entre os diagnósticos diferenciais não apenas permite o reconhecimento precoce, como também viabiliza intervenções terapêuticas capazes de modificar a evolução natural da doença.

## Data Availability

Os conteúdos subjacentes ao texto da pesquisa estão contidos no manuscrito.
