# Supplementary material for: Doença de Fabry de Início Tardio: Desafios Diagnósticos e Evolução Clínica
Source: Arq Bras Cardiol. 2026 Mar 26;123(2):e20250554. [Article in Portuguese] doi: 10.36660/abc.20250554 (PMC13128178; doi:10.36660/abc.20250554)
Supplement: Translation

# Translation: Late-Onset Fabry Disease: Diagnostic Challenges and Clinical Evolution

**DOI:** 10.36660/abc.20250554i

**Authors:** Hernán Patricio García Mejía, Heleuterio da Conceição Nicolau Madogolele, Manuela Cristina Ribeiro Dias Barroso, Matheus Carvalho Alves Nogueira, Kevin Rafael de Paula Morales, Cristhian Espinoza Romero, Luciano Nastari, Fábio Fernandes

**Affiliations:** 1 São Paulo SP Brazil Instituto do Coração do Hospital das Clínicas da Faculdade de Medicina da Universidade de São Paulo, São Paulo, SP – Brazil

**Keywords:** Fabry Disease, Left Ventricular Hypertrophy, Cardiomyopathies, Cardiac Conduction System Disease

## Introduction

Simultaneous cardiac and renal involvement can result from various systemic conditions, such as hypertension, autoimmune diseases, infiltrative diseases, and hereditary syndromes. Fabry disease (FD) is an X-linked genetic disorder caused by a deficiency or absence of the enzyme α-galactosidase A, leading to the progressive accumulation of glycosphingolipids, especially globotriaosylceramide (Gb3), in various tissues.^1^ This accumulation of Gb3 promotes multisystemic dysfunctions, with heterogeneous clinical manifestations that vary according to sex, the degree of residual enzymatic activity, and the form of presentation, classic or non-classic.^2,3^

The estimated incidence of FD ranges from 1 in 40,000 to 1 in 117,000 individuals worldwide.^2^ In Brazil, the epidemiology of FD remains poorly defined due to the rarity of the condition and underreporting of cases, which reinforces the value of clinical reports such as this one.

Although the classic form of the disease manifests early, the late-onset variant is frequently underdiagnosed and is characterized by predominantly cardiac, renal, or neurological involvement in adult patients, usually male. In the cardiology context, the most common manifestation is the non-obstructive hypertrophic cardiomyopathy phenotype, often indistinguishable from other causes without specific investigation.

## Case report

A 71-year-old male patient was referred by the Nephrology team for investigation of left ventricular hypertrophy (LVH). Past medical history: long-standing systemic arterial hypertension (SAH), chronic kidney disease (CKD) requiring dialysis since February 2018, and acute myocardial infarction.

Family history: sudden death of a sister at age 36, father deceased from unspecified heart disease at age 68, and mother at age 60 from an unknown cause. One daughter is alive and healthy.

The initial electrocardiogram (ECG) (2018) showed sinus rhythm, short PR interval (88 ms), left anterior superior divisional block, and signs of LVH (Figure 1-A). Transthoracic echocardiography showed significant LVH, 17 mm interventricular septum, 15 mm posterior wall, non-obstructive pattern (Figures 2-A and 2-B). Catheterization identified an intraventricular gradient of 26 mmHg and triple-vessel coronary artery disease.

**Figure 1:** A) Electrocardiogram showing sinus rhythm, short PR interval (88 ms), left anterofascicular block, and left ventricular hypertrophy. B) Electrocardiogram showing sinus rhythm, first-degree atrioventricular block (380 ms), premature ventricular contractions, and left anterofascicular block. (C) Electrocardiogram showing right bundle branch block pattern and second-degree atrioventricular block Mobitz II.

**Figure 2:** A) Late myocardial enhancement images obtained after gadolinium administration, without evidence of myocardial fibrosis. B) Cine magnetic resonance sequence using SSFP (steady-state free precession) technique, demonstrating concentric increase in left ventricular thickness. C) Native T1 map acquired by MOLLI (Modified Look-Locker Inversion recovery) sequence, demonstrating T1 values of 1119 ms in the interventricular septum and 909 ms in the lateral wall, from regions of interest (ROIs). D) Parametric native T1 map in mid-short axis view, with color-coded scale, visually demonstrating a predominance of elevated values in the septal wall. E) Transthoracic echocardiography showing interventricular septum thickness of 17 mm (red arrow), and the presence of the binary signal (yellow arrow).

Cardiac magnetic resonance imaging (CMR) confirmed symmetrical LVH, preserved LVEF (LV: 74%, RV: 62%), without late enhancement, but with increased extracellular volume (40%; RV <30%) and elevated native T1 (Reference value (RV): 1119 ms; RV 968 ± 21 ms), in addition to an inferolateral area with low T1 (RV: 898 ms).

Given the significant concentric hypertrophy, differential diagnoses such as cardiac amyloidosis and FD were considered. Serum and urinary protein electrophoresis showed a normal pattern, with kappa and lambda light chains within reference values. Enzyme assays revealed reduced α-galactosidase A activity: 0.88 mcmol/L/h (reference range: 1.68–13.63 mcmol/L/h) and LysoGb3 level <0.5 ng/mL (reference range: <2.0 ng/mL). Genetic analysis identified a homozygous mutation in the GLA gene (c.870G>C; p.Met290Ile), a rare variant described in a limited way in the literature, associated with a late-onset phenotype and predominance of cardiac manifestations, confirming the diagnosis of late-onset FD. The patient started enzyme replacement therapy (ERT) with agalsidase beta, 1 mg/kg every two weeks, with a satisfactory initial clinical response.

The patient's daughter also presented the same mutation in heterozygosity, with enzymatic activity within the normal range (2.44 mcmol/L/h). Genetic counseling was provided to other first-degree relatives, with guidance on clinical and molecular screening.

After 4 years of ERT, there was a worsening of functional class, an increase in markers such as BNP/troponin, and palpitations. The ECG showed progressive PR prolongation (88 → 380 ms), first-degree AV block, and frequent ventricular extrasystoles (Figure 1-B). Subsequently, in June 2025, syncopal episodes of the "on-off" type developed. Continuous electrocardiographic monitoring confirmed second-degree AV block, Mobitz II type, and a permanent pacemaker implantation was then indicated and performed. Continuous electrocardiographic monitoring confirmed second-degree AV block, Mobitz II type, and a permanent pacemaker was then indicated and implanted (Figure 1-C).

## Discussion

FD is an X-linked hereditary condition characterized by the accumulation of glycosphingolipids, primarily globotriaosylceramide (Gb3), in various tissues, including myocardium, kidney, endothelium, and cardiac conduction system.^1^ This accumulation results in a heterogeneous clinical spectrum, ranging from severe classic forms to late-onset variants, which are frequently underdiagnosed.^1,2^

In Brazil, the epidemiology of FD is not yet well established due to its rarity, phenotypic variability, and underreporting of cases, resulting in a scarcity of data in national registries. International studies report a prevalence of pathogenic variants of the GLA gene in up to 1% of patients with LVH phenotype.^3,4^ A recent Brazilian study involving hemodialysis patients identified pathogenic variants or variants of uncertain significance in the GLA gene in almost half of the sample, reinforcing the clinical relevance of genetic screening in this context and the need for careful interpretation of rare variants, such as the p.Met290Ile mutation described in the present case.^5^

Classical FD mutations are observed in approximately 1 in 22,000 to 1 in 40,000 men, and the non-classical form in approximately 1 in 1,000 to 1 in 3,000 men and 1 in 6,000 to 1 in 40,000 women.^6^ The classic phenotype, which is more severe, is characterized by the absence or severe reduction of α-Gal A activity, Gb3 accumulation, and early onset of symptoms, presenting mainly in childhood and adolescence. The non-classical, or late-onset, phenotype presents in adulthood, with incomplete systemic involvement and some residual enzyme activity, as in the case of the patient described.

From a structural point of view, Gb3 accumulation affects various types of cardiac cells, such as cardiomyocytes, endothelial cells, smooth muscle, valve fibers, and conducting tissue.^7^ The progressive increase of Gb3 in myocytes leads to the development of LVH and diastolic dysfunction. Involvement of intramyocardial vessels causes structural and functional changes that contribute to the occurrence of myocardial ischemia. The presence of fibrosis and impairment of the cardiac conduction system are related to the emergence of ventricular rhythm disturbances and alterations in electrical conduction. In early stages, a shortened PR interval can be observed, while AV block and bundle branch blocks are more frequent in advanced stages. This highlights the dynamic progression of the changes (Figure 3).^8,9^ The lack of robust data and specific studies on the risk of sudden death and arrhythmias makes risk stratification difficult. A systematic review published in 2017 identified some associated factors, such as advanced age, male sex, LVH, and late enhancement on CMR, but more specific studies are still needed.^6^

**Figure 3:** A) Patient genogram; B) Evolution of patient changes chronologically; HFpEF: Heart failure with preserved ejection fraction; HFrEF: Heart failure with reduced ejection fraction; AV block: Atrioventricular block.

Diagnostic methods include imaging studies such as echocardiography and CMR, enzyme activity tests, genetic testing, family history, clinical evaluation, and tissue biopsies. Differential diagnosis is based on the spectrum of hypertrophic cardiomyopathies. In a recent study involving 5,491 patients diagnosed with LVH and/or hypertrophic cardiomyopathy, the prevalence of pathogenic variants in the GLA gene was 0.93% in men and 0.90% in women.^4^

CMR and T1-weighted imaging (T1-weighted imaging) are fundamental tools in the diagnosis and monitoring of FD. Native T1-weighted imaging measures the longitudinal relaxation time of myocardial tissue without the use of contrast, aiding in tissue characterization. In FD, the accumulation of Gb3 in the myocardium normally leads to a reduction in native T1 values. However, increased hypertrophy, intensification of interstitial fibrosis, and myocardial inflammation are potential mechanisms that explain the phenomenon of T1 pseudonormalization.^10^ This phenomenon, already reported in previous studies,^11,12^ can make the isolated interpretation of T1 difficult. Therefore, the combination of T1-weighted imaging with other imaging modalities and clinical assessments is essential for an accurate diagnosis.^13^

ERT has been shown to slow the progression of heart disease and reduce the incidence of cardiovascular events. In individuals with late-onset FD, it should be considered if there is evidence of renal, cardiac, or neurological damage, even in the absence of typical symptoms.^10^ Long-term studies and registries indicate that ERT can slow disease progression and reduce cardiovascular events. ERT has significantly altered the natural course of FD and improved patients’ quality of life.^8^

Simultaneous cardiac and renal involvement can result from various systemic conditions, which demand extensive consideration of differential diagnoses. Among the most prevalent causes are hypertensive heart disease and kidney disease, which frequently coexist, becoming potential confounding factors in the interpretation of the phenotype.^14,15^ Systemic autoimmune diseases, such as systemic lupus erythematosus and vasculitis, represent other important etiologies, particularly in the presence of clinical and laboratory manifestations indicative of systemic inflammation.^16^ Infiltrative diseases, such as amyloidosis, sarcoidosis, and hemochromatosis, should also be considered, given their ability to cause myocardial dysfunction and progressive renal injury.^17^ In addition, hereditary syndromes, including sickle cell anemia, Alport disease, and some mitochondrial diseases, should be investigated.^18^

Because this is a unique case, there are inherent limitations to its wider applicability. Even so, the report highlights the diagnostic challenges posed by comorbidities and the relevance of genetic screening.

## Conclusion

The non-classical form of FD often manifests insidiously in adulthood, with atypical signs and symptoms that make it difficult to recognize. Cardiac abnormalities, such as LVH, myocardial dysfunction, and conduction system disorders, can occur even in the absence of typical extracardiac manifestations. Furthermore, the presence of common comorbidities, such as hypertension and CKD, can mask the clinical picture and delay diagnosis. Therefore, in-depth etiological investigation is essential in cases of hypertrophic cardiomyopathy in adults. Including FD among the differential diagnoses not only allows for early recognition but also enables therapeutic interventions capable of modifying the natural progression of the disease.

## Data Availability

The underlying content of the research text is contained within the manuscript.
